# Implementing an intervention to reduce use of antibiotics for suspected urinary tract infection in nursing homes – a qualitative study of barriers and enablers based on Normalization Process Theory

**DOI:** 10.1186/s12877-022-02977-w

**Published:** 2022-03-31

**Authors:** Marius Brostrøm Kousgaard, Julie Aamand Olesen, Sif Helene Arnold

**Affiliations:** 1 Department of Public Health, The Research Unit for General Practice and Section of General Practice, University of Copenhagen, Copenhagen, Denmark; 2 Department of Clinical Microbiology, Herlev and Gentofte Hospital, University of Copenhagen, Herlev, Denmark

**Keywords:** Complex intervention, Implementation barriers, Implementation enablers, Nursing homes, Qualitative study, Urinary tract infection

## Abstract

**Background:**

Overuse of antibiotics in the elderly population is contributing to the global health problem of antibiotic resistance. Hence, it is important to improve prescribing practices in care facilities for elderly residents. In nursing homes, urinary tract infection (UTI) is the most common reason for antibiotic prescription but inappropriate prescriptions are frequent. In order to reduce the use of antibiotics for suspected urinary tract infection in this context, a complex intervention based on education as well as tools for reflection and communication had been developed and trialed in a group of nursing homes. The presents study explored the barriers and enablers in implementing this complex intervention.

**Methods:**

After the intervention trial period, a qualitative interview study was performed in six of the nursing homes that had received the intervention. The study included 12 informants: One senior manager, four nurses, six healthcare assistants, and one healthcare helper. Normalization Process Theory was used to structure the interviews as well as the analysis.

**Results:**

The intervention was well received among the informants in terms of its purpose and content. The initial educational session had altered the informants’ perceptions of UTI and of the need for adopting a different approach to suspected UTIs. Also, the study participants generally experienced that the intervention had positively impacted their practice. The most important barrier was that some of the interventions’ clinical content was difficult to understand for the staff. This contributed to some problems with engaging all relevant staff in the intervention and with using the observation tool correctly in practice. Here, nurses played a key role in the implementation process by regularly explaining and discussing the intervention with other staff.

**Conclusion:**

The results suggest that it is possible to implement more evidence-based practices concerning antibiotics use in nursing homes by employing a combination of educational activities and supportive tools directed at nursing home staff.

**Supplementary Information:**

The online version contains supplementary material available at 10.1186/s12877-022-02977-w.

## Background

Antibiotic resistance is a serious global health challenge caused by excessive use of antibiotics [1], and overuse of antibiotics to the elderly population is an important part of this problem [2]. Consequently, improving prescribing practices in health care has become a priority, especially in care facilities for elderly residents [2, 3]. In nursing homes, urinary tract infection (UTI) is the most common reason for antibiotic prescription but inappropriate prescriptions are frequent [2, 4, 5]. While several factors influence the prescribing process, two common misconceptions among healthcare staff seem to be of particular importance to the overuse of antibiotics in relation to suspected UTI in nursing home residents [6, 7]. First, nonspecific behavioral symptoms in the elderly are often interpreted as signs of UTI [8] although guidelines do not recommend antibiotic treatment for such symptoms [9]. Considering that nursing home residents often are medically complex, there can be several possible causes for changes in their behavior. Second, when health professionals suspect a UTI, it is common to do some form of urine test and dipstick testing and culture is by far the most prevalent in the Danish nursing home setting. In the elderly, bacteria in the urine without symptoms from the urinary tract are common [9]. This is called asymptomatic bacteriuria and should not be treated with antibiotics [10]. However, the presence of bacteriuria can lead healthcare staff to assume that UTI is the cause and therefore, frequent use of testing without due considerations becomes a source of overtreatment [11].

In many nursing home settings, physicians do not usually see the patient directly when making decisions about prescribing antibiotics [12, 13]. It is the staff who decides when it is necessary to contact a physician and it is the staff who conveys the information about the residents’ condition to the physician. Therefore, nursing home staff play a central role in decision processes concerning prescriptions of antibiotics to nursing home residents [14–16]. Consequently, one way to reduce inappropriate prescriptions of antibiotics is to make sure that nursing staff have sufficient knowledge of UTI among the elderly, that they are able to make relevant clinical observations, and that they know when and how to communicate these observations to the prescribing physician [2, 3, 15]. This was the rationale of an intervention in Danish nursing homes which aimed at reducing prescriptions by providing knowledge and tools to support increased reflection among the nursing home staff and to support communication with general practice about suspected UTI [6]. The intervention was designed in a tailoring process and its effectiveness was tested in a cluster randomized controlled trial [6, 7, 17]. Since implementation of complex interventions is often difficult and since few implementation studies of interventions to reduce antibiotics use for suspected UTI have been conducted in nursing homes, we performed a qualitative study of the barriers and enablers to implementing the intervention in the nursing homes during the trial period.

### Setting and intervention

#### The setting

In Denmark, the provision of nursing home services is the responsibility of the municipalities. Most nursing homes are public while some are private (but also operate under public regulations). Nursing homes employ several types of care staff of which the majority are healthcare helpers and healthcare assistants. Healthcare helpers have attended education for 19 month and provide daily care and practical assistance to residents (access to this education requires a graduation exam from elementary school). Healthcare assistants have additional 20 months of education and provide personal support, nursing care, and sometimes also medication dispensing and administration. Most nursing homes also have a small number of nurses employed or associated. Nursing education lasts 3.5 years at minimum and access requires a graduation exam from high school (or similar qualifications). Nurses in nursing homes are typically responsible for coordination of care, documentation, supervision, prevention, quality improvement, and planning of continuing educational activities. A nurse usually gets involved when the condition of a resident deteriorates.

#### The intervention

Details concerning the rationale of the intervention, the development of the intervention, and the results from the effectiveness trial are reported elsewhere [6, 7, 17]. The intervention consisted of an introductory educational session and two paper-based tools to be used by the staff when they suspected UTI in a resident:

1. a tool for observation and reflection (henceforth the reflection tool).
2. a tool for communicating with the GP (henceforth the communication tool).

Both tools can be found in a previous paper on the development of the intervention [7]. The reflection tool had three sections that mirrored the purpose of the tool [7]:

- a section for observing signs and symptoms with the purpose of supporting staff awareness of symptoms of UTI as well as symptoms that could point in other directions than UTI.
- a section with a flowchart to indicate the likelihood of UTI in the case at hand.
- a section which encouraged collective reflections among staff about the case in question.

The tool had been developed on the basis of the Loeb Minimal Criteria for ordering a urinary culture [18]. As with the Loeb Criteria, the purpose of the reflection tool was to avoid unnecessary suspicion of UTI due to asymptomatic bacteriuria but also to make the staff able to consider and potentially exclude other causes (somatic and non-somatic) if the resident showed nonspecific behavioral symptoms.

The purpose of the communication tool was to support the staff in delivering structured and relevant clinical information when they found it necessary to contact general practice. The communication tool was based on the principles of the ISBAR (or SBAR) concept for communication in the healthcare sector (ISBAR: Identification, Situation, Background, Assessment and Recommendation) [5, 6, 19].

In the interactive educational session, the staff received education in UTI with a focus on how to assess nonspecific symptoms, how to distinguish between UTI and asymptomatic bacteriuria, and the caveats of using urine tests. So while the intervention did not aim to discourage testing as such, it did aim to discourage what might be called a more test-focused approach to diagnosing UTI where testing is performed before clinical symptoms from the urinary tract has been ascertained, and before other possible causes of nonspecific behavioral symptoms has been ruled out.

Staff were also introduced to the tools of the intervention with practical case-based exercises. The duration of the educational session was 75 min. The same educational session was carried out 2–5 times at each nursing home depending on the number of physical localities of the nursing home. The nursing homes were also provided with posters informing staff about the intervention.

The intervention was delivered in 2018 (October and November) to 11 nursing homes in the Capital Region of Denmark (with 11 nursing homes in the control group). Afterwards, the nursing homes should use the knowledge and tools of the intervention in the trial period from 1 December 2018 to 31 March 2019. Characteristics of nursing homes (and nursing home residents) in the intervention have been described elsewhere [17]. SHA was the principal investigator on the development of the intervention and on the design and execution of the randomised control trial. MBK and JAO contributed to the development of the intervention and JAO also assisted with the execution of the randomised control trial.

## Methods

### Study design

The study was a qualitative interview study based on Normalization Process Theory (NPT). As a theory, NPT was designed to study efforts to implement and sustain (normalize) complex interventions in health care settings and understand the factors that contribute to success or failure in these processes [20, 21]. According to NPT, the most important barriers and enablers when implementing complex interventions are related to four different constructs:

1. *Coherence* refers to the importance of securing that participants have a shared and sufficient understanding of the intervention, how it differs from previous practice (differentiation), and that they view the intervention as a valuable contribution to their practice.
2. *Cognitive Participation* refers to the importance of creating and sustaining commitment and engagement around the intervention.
3. *Collective action* refers to the importance of making the intervention manageable in practice (*interactional workability*) and of establishing trust around the intervention in the networks of professionals involved in and affected by the intervention (*relational integration*). This construct also covers how the intervention fits with existing rules and technologies, how resources are allocated to support implementation (*contextual integration*), and how work is delegated, so that the tasks involved in implementing the intervention matches the competences of the people designated to perform them (*skill-set*).
4. *Reflective monitoring* refers to how professionals assess (formally or informally) the consequences of the intervention and how these assessments affect the implementation process, e.g., if they lead participants to adjust or transform the intervention.

### Nursing homes and study participants

Eleven nursing homes in the Capital Region of Denmark received the intervention during the trial period.

Since we did not have resources to include all the nursing homes in the qualitative study, we excluded nursing homes that had self-ownership (*n* = 2) (thereby reducing some of the variation in institutional context and simplifying the analysis), nursing homes where the implementation process had been delayed due to a high work load and a shortage of staff (*n* = 2) (since we wanted to interview staff about their experiences with using the tools), and nursing homes that did not have a regular GP (*n* = 1) (since at a later stage we planned to interview the regular GP at the included nursing homes). The six remaining nursing homes all agreed to participate.

From each of the included nursing homes we aimed to interview one manager *or* one nurse who had been closely involved in the implementation process as well as one health care assistant who had used the reflection tool in practice. We initially contacted the management of each nursing home and asked them to identify two potential informants based on these criteria, and these informants were subsequently invited to participate in the study. All the invited informants agreed to participate. At one of the nursing homes, a health care helper who had also been involved in the implementation process offered to participate in the interview with the health care assistant which we agreed to. Hence, the study included 12 informants (11 females and one male) from the six nursing homes: One senior manager, four nurses (one of the nurses was employed at two of the nursing homes); six healthcare assistants, and one healthcare helper. Informed consent was obtained from all participants.

### Data collection

The interviews were semi-structured. The interview guide was inspired by NPT (see specific questions in Additional file 1). Questions were related to the four dimensions of NPT with attention to implementation barriers and enablers:

- Coherence: Initial response to the goals and principles of the intervention, perceptions of the intervention compared with usual practice, comprehensibility of the intervention and its components, actions to support comprehensibility.
- Cognitive participation: The engagement of management and staff, delegation of responsibility for the implementation process.
- Collective action: Experiences with using the intervention tools in practice, division of work in relation to using the tools, reactions of residents and relatives, collaborative relations with external actors.
- Reflexive monitoring: Perceptions of the consequences of adopting the intervention e.g., changes in terms of knowledge and practice concerning suspected UTI (including collaboration with other health care actors).

In the interviews we focused on implementation in regards to those components of the intervention which could be relevant to implement after the trial in a non-research context – and not on issues and components that were only related to the trial (such as an extra registration form).

Interviews were carried out at each of the nursing homes in April and May 2019, i.e. after the trial period had ended. The interviews were audio recorded and lasted 20–50 min (depending on the informants’ experiences with intervention use and the implementation process as a whole). All interviews were individual interviews (except one where two informants were present), and the interviews were carried out either by an experienced qualitative researcher (MBK) or a research assistant (JAO) except for the first interview where both interviewers participated.

### Analysis

All interviews were transcribed. Initially, two of the authors (MBK and JAO) read all of the transcribed interviews to obtain an overview and a familiarity with the content of each of the interviews. Subsequently, we presented our initial understandings of the data to each other and discussed preliminary themes related to the aim of the study. The NPT constructs had already been used to guide the interviews, and we considered that it was also feasible to use NPT as a coding framework thereby taking a somewhat deductive approach to the analysis [22]. Having first coded two interviews using the NPT constructs, MBK and JAO compared codes and clarified emerging questions. This process of coding, comparing and discussing continued until all interviews had been coded. Interviews were coded in NVivo 12. Then, all the coded material was inspected, condensed and organized to enhance overview. Subsequently a draft of the analysis based on NPT was discussed and refined by the authors. The professional backgrounds of the researchers – social science (MBK), public health (JAO) and medicine (SHA) – offered different perspectives on the data. Quotations are used illustratively. All methods were carried out in accordance with relevant guidelines and regulations.

## Results

First, we present the results on the barriers and enablers in relation to the implementation of the reflection tool and the knowledge related to – and embedded in – this tool. The presentation is structured along the four dimensions of NPT. As it turned out, the third element of the intervention, the communication tool, had rarely been used by the staff, and lastly, we explain why this was the case.

### Coherence

The overall goal of reducing antibiotics use was well accepted by the informants in our study who were positive about the knowledge and the approach promoted by the intervention and communicated to the staff at the educational workshop:

*The amount of antibiotics that we use without thinking about it, sometimes without a real indication of urinary tract infection […] that was a wake-up call for me [Healthcare assistant, Informant #12]*

Further, the informants were able to see how the intervention differed from usual practice and this difference was described to be positive:

*The idea that you suspect urinary tract infections is new. It’s a change of the mindset we have had for many years – a mistaken mindset I would say […] [previously] we would think that if there are bacteria in the urine then it’s a urinary tract infection and then you have to treat that with Selexid [pivmecillinam]. But no! You can have bacteria in the urine without having an infection. That was an eye-opener even for me as an experienced nurse [Nurse, Informant #9]*

*The most important message, as far as I remember, was that in order to avoid that a possible infection results in [antibiotic] treatment there are some things that you can do, so that not everyone get these antibiotics [Healthcare assistant, Informant #7]*

However, as also seen in the first quotation, the intervention was not just about ‘adding’ something new to the existing practice – it was also about unlearning and de-implementing existing beliefs and practices. At a few of the nursing homes, some staff had questioned the new and more restrictive approach of the intervention due to concerns of undertreatment. Here it was important to convince the staff of the potential value of the intervention:

*The staff was very concerned when we started this process. They also expressed this during the workshop. Fortunately, there was a bit of time – perhaps not quite enough in my opinion – so that they could voice their concerns. It’s a process where they have to change their thinking from ‘we know that it’s always a urinary tract infection’ to ‘sometimes it might be something else that we have missed’. But I was in total agreement from the beginning.” [Nurse, Informant #3]*

Furthermore, many of the health care assistants and health care helpers had initially felt that the tool was somewhat overwhelming and difficult to understand in details due to parts of the terminology. Particularly, terms like ‘delirium’, ‘non-specific changes’, and ‘newly emerged symptoms’ created difficulties:

*Interviewer: Do you remember your thoughts when the observation/reflection tool was presented?*

*Health care assistant (Informant #7): Oh my God! [laughing]*

*Heath care helper (Informant #8): What’s this?! And how do we start to use it?*

The challenges with understanding the tool among relevant staff in the organizations was exacerbated by the fact that not all staff had participated in the educational workshop (including some of the staff with implementation responsibilities). Instead, they had been introduced to the intervention by their team leader (or another colleague), but this introduction did not include the exercises from the workshop nor necessarily all of the knowledge provided at the workshop. Problems with understanding the details of the tool became particularly apparent when staff first attempted to use the tool in practice, and here the nurses (and some of the assistants) played a central role in explaining to their colleagues how to apply the tool when interacting with the residents (see the section below on collective action).

### Cognitive participation

According to the informants, staff engagement in the intervention had varied at the wards of the nursing homes; at some wards the reflection tool had been used regularly by most staff when suspecting an UTI, at other wards the tool had primarily been used by a few engaged staff. Various challenges with engaging staff in the use of the tool were mentioned. First, according to the informants some staff had been uncomfortable with using the tool due to difficulties with understanding (cf. coherence). Second, some staff found it difficult to remember to use the tool during a busy workday with many different tasks and several other forms to fill out:

*I had to make myself remember to use the form. That was the most difficult. Sometimes I just started to act [and then]: ‘oh no, I have to fill out the form!’ [Health care assistant, #12]*

Drivers for creating cognitive participation among the staff were the enrolment work of managers and nurses (and sometimes also other designated key persons among the staff). At some nursing homes, management regularly reminded staff to use the intervention and the informants experienced that this had strengthened implementation. At other places, management was less involved in the implementation process, but regardless of the level of direct management involvement, much of the responsibility for ensuring implementation was delegated to one or two nurses at each of the nursing homes. These nurses were motivated to drive the intervention forward because they found the new approach to be in line with their professional role; some were already employed in positions where they were responsible for quality improvement and educational activities, and they believed in the value of a more systematic and knowledge-based approach to practice (cf. coherence):

*“It [the reflection tool] quickly clarifies whether UTI is likely or not, and it is not so much based on what someone just happens to think, it is based on this [what the staff have noted on the reflection tool] […] and then we can talk about that” [Nurse, #5]*

When the nurses (and/or local team leaders) used the daily triage meetings to talk about the intervention and reminded staff to use the reflection tool, this seemed to support engagement with the intervention among the staff:

*We have daily triage meetings where we [the assistants] meet with a nurse, and there we use it [the reflection tool] a lot (Healthcare assistant, #6)*

At the meetings, the nurses both tried to create awareness, a sense of obligation, as well as increased capacity for using the tool:

*I sat down with the staff many times and when they said ‘oh, we just forgot [to use the reflection tool]’ then I said: ‘okay, let’s look at it and go through this flow chart and think about what we shall do’. […] I have also explained the tool many times. [Nurse, #11]*

Some nurses also tried to increase engagement among staff by making weekly visits to the wards where they reminded staff to use the tool and inquired about cases where the tool could have been used. This required time and energy and it was a challenge to reach all staff in the organizations:

*I had forgotten how much it takes to be present at every ward and look the staff in the eyes and say: ‘remember our focus’ […]. At the nursing homes there are a lot of staff […] and, so I was a bit surprised how difficult it actually was to reach everybody” [Nurse, #3]*

### Collective action

The main challenge with using the reflection tool (correctly) in daily practice was related to problems with understanding the tool and its terminology (cf. coherence). To deal with this issue it proved important that individual staff did not use the tool alone but always engaged in a dialogue with a colleague about the specific case when UTI was suspected (and the tool itself also suggested this in the reflection section). Here, the nurses played an important role in helping the other staff with using the tool. Thus, the nurses did not usually apply the tool ‘at the bedside’ themselves but had more of a supervisory role. This division of work followed the nursing homes’ overall approach to implementing the intervention which was to integrate the use of the reflection tool into the existing organization of work where it was the healthcare assistants and the healthcare helpers who interacted with the residents on a daily basis. Therefore, it was the healthcare assistants (or sometimes the healthcare helpers) who first applied the tool when they suspected UTI in a resident. The subsequent involvement of a nurse (often at the daily triage meeting where the nurses promoted the use of the tool) seemed to foster more reflections and a better capacity among staff for using the tool in practice:

*“I think it’s about using it repeatedly and talk about it in the group. And the nurse has been involved in this as a key person. I think we have become more familiar with the tool than we were in the beginning. It was difficult in the beginning…” [Healthcare assistant, #10]*

Most of the informants experienced that the health care assistants were able to apply the tool in practice with some supervision from a nurse, particularly in the beginning. As for the healthcare helpers (who had the shortest education), the informants considered that these should only use the tool in close collaboration with an assistant (and again with the subsequent involvement of the nurse). In two of the nursing homes, it had been decided that the healthcare helpers should not use the tool but leave it to the healthcare assistants and the nurses. However, a few of the nurses believed that the health care assistant also needed further education in diseases among the elderly before they were able to use the tool properly. One of the nurses described that she had often had to correct the staffs’ use of the tool:

*“…for a helper or an assistant alone on the night watch, it is too complex for their level of knowledge. I have experienced that they make some assessments which are outside their area [of competence], with delirium for example. I have seen several cases where they note that the resident has acute delirium… but when I follow up by contacting the staff it turns out that is has nothing to do with delirium” [Nurse, #3]*

Concerning interactions with the residents, a few informants suggested that it was more difficult to apply the tool when dealing with residents with dementia since these could not explain their symptoms as well as other residents:

*Here at the somatic ward, we have residents who can express their symptoms. So, we have probably been a bit better at using it than at the dementia ward [healthcare assistant, #12]*

Two of the nursing homes had made a targeted effort at informing the residents and their relatives about the intervention (e.g., via newsletters, meetings) while the rest of the nursing homes had taken a more ad hoc approach by mainly informing about the intervention if any questions arose about the handling of specific cases of suspected UTI. According to the informants, many residents and relatives were not aware that a new intervention had been introduced, but those who did become aware generally responded positively to the new approach:

*When I talked to the residents about the project, some of them said: ‘well, actually I have also wondered why I am always treated for urinary tract infection even though it doesn’t burn or anything’ […]. So, they were also wondering a bit. [Nurse, #3]*

However, some of the informants mentioned that relatives had occasionally insisted on treatment with antibiotics when suspecting UTI even though the staff had decided to wait. While such behavior went against the objectives of the intervention, the informants experienced that this problem could usually be managed through information and dialogue:

*”…we have experienced that a family member has taken matters in her own hands and acquired a prescription for antibiotics. But it actually only happened a few times. Because I think that when you talk reason, they can see that it makes sense not to start a treatment without thinking it through” [Nurse, #5]*

#### Relations with external actors

During the trial, the informants had different experiences with integrating the new approach of the intervention when collaborating with other actors about residents with suspected UTI. In some of the nursing homes, the informants had experienced that external actors from the hospital sector (especially psychiatry) or the municipality (e.g., the external dementia nurses) had a more test-focused approach to antibiotic treatments, which was not congruent with the approach promoted by the intervention:

*“If we have a resident who becomes agitated then the first question which the dementia consultant [a nurse] asks is: ‘did you take a dipstick?’. If we contact psychiatry […] then that’s also the first thing they ask us. So, there is still some way […] so it can be a dilemma” [Manager, #1]*

In such cases, the staff would explain the rationale behind the more restrictive approach of the intervention and this was usually accepted by these collaborative partners:

*Nurse [#5]: […] in psychiatry they ask for it [a dipstick test result] every time*

*Interviewer: And how do you respond to that?*

*Nurse [#5]: Well, then we say that we do not use dipsticks. They can have a urine sample at the general practice if they insist […]. There is still a big myth in psychiatry that when it comes to the elderly and confusion and behavioral changes, then it must be a urinary tract infection. They want all somatic issues to be solved before they can start their treatment […] I have not experienced negative reactions. They may ask questions as to why we won’t do a dipstick test, but when I explain it, they understand.*

Concerning general practice, the informants experienced that the approaches of the general practitioners (GPs) to UTI influenced the interventions’ impact on staff behavior at the nursing homes but also that these approaches varied widely among the GPs. When the approach of the GP was in congruence with the principles of the intervention this was an important driver of implementation:

*He [the regular GP at the nursing home] was very good a supporting this by saying: ‘I think we should [first] try with a fluid balance chart’ or ‘I think we shall wait and see, and then we can discuss it on Monday if you are still concerned’. That had a great effect on the staff, so that they dared to use this [Nurse, #3]*

Contrary, the more test-focused approach of other GPs could work against the principles of the intervention:

*“When we contacted the GP, they asked us to make a dipstick and sometimes if the resident has a history with UTI they ask for a urine sample to be tested. We have done that but only when the GP asks us to do it.” [Health care assistant, #12]*

Since it was assumed beforehand that the GPs would usually be the most important of the external collaborators concerning suspicions and decisions about UTI, the local GPs had been informed about the intervention by letter. Further, GPs serving as regular nursing home physicians had been invited to the workshop but had not participated in the workshops held at the six nursing homes that were part of this study.

### Reflexive monitoring

In spite of the implementation challenges mentioned above, the informants were generally positive about the intervention because they experienced that it had a positive impact on their approach to suspected UTIs. First and foremost, the informants emphasized that by providing new knowledge and specific guidance, the intervention had increased the staffs’ ability and confidence – and thereby also their tendency – to reflect about situations where UTI was suspected. This included considering whether the observed signs and symptoms could point in other directions than UTI:

*So, the assistants […] have become better at saying that it doesn’t have to be an infection just because there are bacteria [in the urine]. That’s a huge difference compared to before [the intervention] [Nurse, #9]*

*You reflect more, you get this dialogue [with a colleague] rather than being quick to reach for the phone [to call the GP] and get some medicine for urinary tract infection. You get a more professional talk where you go into details, and that requires you to think more […] to be curious” [Healthcare assistant, #10]*

As indicated by these quotes, the increased inclination to reflect about suspicions of UTI had practical implications. At the nursing homes this could involve one (or more) of the following changes: less use of dipstick testing, adopting more of a wait-and-see approach with increased use of preventive measures focused on residents’ hygiene and fluid intake, and/or reducing the number of calls made to the GP for prescriptions of antibiotics:

*Previously there was this tendency just to say: ‘well, let’s get a dipstick test’. Now there is a bit more reflection about ‘why and what should I actually choose to do here?’ [Healthcare assistant, #4]*

*…we have started to think that it may be other things than a UTI, and we have increased our use of fluid schedules a lot, and sleep cycle, and stuff like that, instead of just sending in a urine sample” [Healthcare assistant, #6]*

While these types of changes were important in relation to the overall objective of the intervention, they were not regarded to be completely implemented in the nursing homes due to the challenges with getting all staff to use the reflection tool systematically:

*…we have become better, but there is still a long way to go. We still need to improve, but we are getting there […] it has been an eye-opener [Manager, #1]*

### Why the communication tool had rarely been used

According to our informants, the communication tool for contacting the GPs had rarely been used by the staff. While the purpose and content of the communication tool was easy to understand and there was no opposition to the tool as such, the limited use of the communication tool could be ascribed to the following factors: First, in terms of coherence (differentiation), the informants did not perceive the communication tool to implicate a real change compared to usual practice, since many were already familiar with the principles of the ISBAR (via previous training) and believed that they already applied the central aspects of the concept in their usual practice. Second, in terms of interactional workability, when staff had already registered their observations in the reflection tool, some perceived it as less relevant and a bit more troublesome also to use and fill in the communication tool. Instead, they could just use the completed observation when passing on information to the GP:

*we are used to working in this way when we contact the doctor. I think it’s superfluous as an addition to the [observation/reflection] schema*. *(Health care assistant, #4)*

*[when] we used the observation tool and noted all the information, it [the communication tool] was perhaps less necessary (Nurse, #11)*

Third, in terms of reflexive monitoring, the few informants who had used the communication tool did not experience that it had made much difference in their communication with the GP. For the informants, the most important aspect of the intervention was the new knowledge and the more systematic approach to observation and reflection. Finally, in wards where a more reflexive and restrictive approach had reduced contacts to the GP, the number of occasions for using the communication tool had, naturally, also been reduced:

*It has not been necessary to use the ISBAR a lot because in most cases we have decided not to contact the doctor since we already managed to deal with the issues [Nurse, #5]*

## Discussion

The intervention was generally well received among the informants in terms of its overall purpose (addressing the problem of antibiotics overuse) and content (knowledge about how to recognize and how to prevent UTI along with a tool to support reflection when suspecting UTI). The initial educational session had altered the informants’ perceptions of UTI and of the need for adopting a different approach to suspected UTIs (coherence). Also, the study participants generally experienced that the intervention had positively impacted their practice (reflexive monitoring). Still, the study identified barriers to implementing the intervention in the nursing homes. The most important barrier was that some of the interventions’ clinical content was difficult to understand for staff (coherence). This contributed to problems with engaging all relevant staff in the intervention (cognitive participation) and to problems with using the reflection tool correctly in practice (collective action). The nurses responsible for implementation played a key role in addressing these issues of coherence, cognitive participation, and collective action by drawing attention to the intervention regularly and by explaining and discussing the intervention with the staff – both ad hoc and at regular meetings.

There are many obstacles to optimizing the decision process involved in the use of antibiotics at nursing homes [12, 23–25]. In our study of a complex intervention for reducing use of antibiotics when suspecting UTI, the most important implementation challenge seemed to be to create a solid understanding of the reflection tool among the staff, particularly among the nurse assistants and helpers. In a recent intervention study by Potter et al. [26], some questions were also raised about the qualifications of some care home staff to use a decision-making algorithm for reducing antimicrobial prescribing. And in a study of implementing a care pathway for elderly patients, Røsstad et al. [27] reported that nursing assistants were uncertain about how to “to observe, assess, act, and document issues on the checklists”. In the context of the present study, the process of developing the intervention prior to the trial had indicated that understanding the reflection tool and the knowledge embedded in the tool could be a challenge for nursing home staff, and the reflection tool had consequently been through several adjustments to increase user-friendliness [7]. Also, the initial educational session – which introduced the tool and gave staff the opportunity to try it out and discuss various questions and concerns – had been well received by the staff. Still, the challenges with understanding the tool during the trial suggests that the educational element might be developed further with an increased focus on hands-on use during introduction in future attempts to implement the intervention more widely. Since such educational sessions are resource consuming in terms of staff attendance, it could be considered to prioritize the reflection tool at the expense of the communication tool since the reflection tool was much more important in terms of influencing practice at the nursing homes.

As mentioned above, the nurses played a key role in creating understanding (and engagement) among the staff during the trial and this finding aligns with the increasing interest in exploring the potential of allying with nurses in antibiotic stewardship interventions [14]. Recent studies have also pointed to the important work of nurses in providing guidance and supervision to other staff during implementation of complex interventions in nursing homes [26, 27]. Also similar to our results, Røsstad et al. [27] found that implementation was facilitated when nurses used existing meetings to increase attention to – and involvement with – the intervention. Such ways of engaging staff were also used by the implementation champions in Potter et al. [26].

Pressure from relatives of residents is a known driver for antibiotics prescribing in nursing homes [14, 15, 23], and can thus be a barrier to the implementation of antibiotic stewardship interventions [25, 26]. While some informants in our study did mention pressure from relatives as a complicating factor, the staff had usually been able to deal with this. As also suggested by others [25, 26], it is important that individual staff (perhaps designated based on social skills and/or experience) are prepared to manage relations with relatives when introducing a more restrictive approach to use of antibiotics. Our results also suggest that implementation was strengthened when nursing home staff were able to explain the rationale for the more restrictive approach in their interactions with external health professionals such as dementia nurses, psychiatrists and GPs. The GPs were the most influential collaborators concerning prescribing decisions but the nursing home staff experienced that the views and approaches of individual GPs varied. Variations in GPs views and approaches to using antibiotics have been reported in previous research which has suggested that differences in risk perceptions may lead to differences in prescribing approaches [15, 28]. Generally, since the views and approaches of external collaborating health professionals to using antibiotics may vary significantly, antibiotic stewardship interventions in nursing homes should consider strategies for handling relations with external collaborators (see Table 1 for an overview of key findings and implications).

**Table 1:** Summary of key findings and their implications for subsequent efforts to optimize and implement the intervention

**NPT construct**	**Key findings**	**Implications for subsequent attempts to develop and implement the intervention**
Coherence	The informants were generally positive about the goals and principles of the intervention. However, they also reported that some staff had been concerned about undertreatment (as a consequence of a more restricted approach), and there were difficulties understanding the clinical terminology of the reflection tool	The educational element of the intervention should be developed further to increase acceptance and understanding of the reflection tool. This may be done through an increased focus on a) explaining key concepts, b) reserving more time for discussing potential concerns of staff, and c) reserving more time for hands-on training Increased time for these activities during the educational session may be found by removing the communication tool from the intervention (cf. below)
Cognitive participation	Staff engagement in the intervention had varied and challenges with engagement included: - Feeling that the reflection tool was a bit overwhelming due to issues of understanding - Remembering and prioritizing the tool during a workday with many other tasks - Reaching all staff in the organization Nurses (and/or local team leaders) used routine triage meetings to increase engagement among staff	It is important to ensure that change champions (e.g., nurses or qualified nurse assistants) understand the intervention and regularly have opportunities for explaining the intervention to staff and encourage its use
Collective action	Using the reflection tool correctly could be a challenge for staff due to difficulties with understanding all aspects of the tool. The involvement of a nurse (often at routine meetings) seemed to foster more reflections and a better capacity among staff for using the tool in practice	Routine meetings could be used to ensure that staff regularly discuss (with each other and with nurses or other change champions) how to apply the reflection tool correctly in specific cases
	Resistance from relatives to the intervention’s more restrictive approach to antibiotics had been infrequent and could usually be managed through information and dialogue Health professionals from collaborating organizations were often perceived to have a more test-focused approach than that promoted by the intervention, but they generally accepted the intervention approach when nursing home staff explained the rationale	(Designated) staff should be prepared to manage relations with relatives and health professionals outside the organization when introducing a more restrictive approach to the use of antibiotics
	The approaches of GPs to dealing with suspected UTI was a mediating factor which could either support or dampen the implementation of the intervention principles at the nursing homes	Local physicians responsible for prescribing medication should be informed about the intervention (and involved actively if possible) to increase the likelihood that their attitudes and actions align with the principles of the intervention
Reflexive monitoring	Informants found that the intervention had increased the tendency to reflect on situations where UTI was suspected and that this had positive consequences in terms of: - reduced use of dipstick testing, - employing a wait-and-see approach with increased use of preventive measures focused on residents’ hygiene and fluid intake, and/or - reduced number of calls to the GP for prescriptions of antibiotics	In spite of some implementation challenges, the results suggest that it is possible to create more evidence-based practices concerning antibiotics use in nursing homes by employing a combination of educational activities and supportive tools
	While the educational session and the reflection tool had influenced knowledge and practice at the nursing homes, the communication tool had rarely been used since staff did not perceive a real need for the tool when communicating with general practice (and since the intervention had sometimes reduced the perceived need for contacting general practice)	It should be considered to remove the communication tool from the intervention so that the time spent on introducing it could instead be used on introducing the reflection tool (since this was a much more important element in the intervention)

### Strengths and limitations

While we included more than half of the nursing homes from the intervention group in this qualitative study, it could be considered a limitation that we only included two informants from each of these nursing homes (due to resource considerations). Particularly, including additional healthcare assistants and healthcare helpers from each of the nursing homes would have generated more knowledge on the details of implementation at individual wards as well as on the variations in use between wards and between individual staff (and hence perhaps have enriched the analysis of the theoretical constructs).

Further, the identification of participants by the nursing home managers might have resulted in the most positive and engaged staff being included in the study. This could mean that the study provides a too optimistic view of the implementation challenges. On the other hand, the selection procedure ensured the inclusion of informants who had practical experience with the tool. And although the informants were generally positive in their assessments of the intervention, they were also critical about some aspects as have been reported in the paper.

We employed NPT, a widely used implementation theory, to shape the interview guide and structure the analysis. NPT seemed well suited for ensuring that important implementation issues were covered at the interviews. During the analysis, applying the theoretical concepts of NPT to the empirical material was more challenging due to problems with overlap between the concepts of cognitive participation, collective action, and coherence. For example, the work initiated by the nurses at the regular meetings could be categorized under collective action since it was about integrating the tool into practice and building confidence in the tool by using it together to reflect on the condition of residents. At the same time, this work of enactment and operationalization involved clarifications on how to understand the terminology of the tool (i.e. coherence), and further, applying the tool during the meetings was also a way to create and sustain engagement with the tool (i.e. cognitive participation). This illustrates how issues of understanding, engagement and enactment/use can be closely entangled in practice. Analytical challenges with overlap between NPT constructs have also been reported in other studies [29–32].

## Conclusion

In spite of the challenges encountered during implementation, the results of this study suggest that it is possible to create more evidence-based practices concerning antibiotics use in nursing homes by employing a combination of educational activities and supportive tools. The results also suggest that particular attention should be given to ensure that staff understands the key principles and terms implicated in this kind of intervention, and to ensure that staff can try out the new the knowledge and tools in practice in the presence of motivated and competent colleagues designated to play a supportive role in the implementation process.

## Supplementary Information

**Additional file 1.**

## Data Availability

The anonymised transcribed interviews from the current study are available from the corresponding author upon reasonable request.

## Abbreviations

GP: General Practitioner
NPT: Normalization Process Theory
UTI: Urinary tract infection

## References

[1] Spellberg B, Guidos R, Gilbert D, Bradley J, Boucher HW, Scheld WM, Bartlett JG, Edwards J (2008). Infectious Diseases Society of America. The epidemic of antibiotic-resistant infections: a call to action for the medical community from the Infectious Diseases Society of America. Clin Infect Dis.

[2] Crnich CJ, Jump R, Trautner B, Sloane PD, Mody L (2015). Optimizing Antibiotic Stewardship in Nursing Homes: A Narrative Review and Recommendations for Improvement. Drugs Aging.

[3] Katz MJ, Gurses AP, Tamma PD, Cosgrove SE, Miller MA, Jump RLP (2017). Implementing Antimicrobial Stewardship in Long-term Care Settings: An Integrative Review Using a Human Factors Approach. Clin Infect Dis.

[4] Nace DA, Drinka PJ, Crnich CJ (2014). Clinical uncertainties in the approach to long term care residents with possible urinary tract infection. J Am Med Dir Assoc.

[5] McMaughan DK, Nwaiwu O, Zhao H, Frentzel E, Mehr D, Imanpour S, Garfinkel S, Phillips CD (2016). Impact of a decision-making aid for suspected urinary tract infections on antibiotic overuse in nursing homes. BMC Geriatr.

[6] Arnold SH, Jensen JN, Kousgaard MB, Siersma V, Bjerrum L, Holm A (2020). Reducing Antibiotic Prescriptions for Urinary Tract Infection in Nursing Homes Using a Complex Tailored Intervention Targeting Nursing Home Staff: Protocol for a Cluster Randomized Controlled Trial. JMIR Res Protoc.

[7] Arnold SH, Olesen JA, Jensen JN, Bjerrum L, Holm A, Kousgaard MB (2020). Development of a Tailored, Complex Intervention for Clinical Reflection and Communication about Suspected Urinary Tract Infections in Nursing Home Residents. Antibiotics.

[8] Kistler CE, Zimmerman S, Scales K, Ward K, Weber D, Reed D, McClester M, Sloane PD (2017). The antibiotic prescribing pathway for presumed urinary tract infections in nursing home residents. J Am Geriatr Soc.

[9] Nicolle LE, Gupta K, Bradley SF, Colgan R, DeMuri GP, Drekonja D, Eckert LO, Geerlings SE, Köves B, Hooton TM, Juthani-Mehta M, Knight SL, Saint S, Schaeffer AJ, Trautner B, Wullt B, Siemieniuk R (2019). Clinical Practice Guideline for the Management of Asymptomatic Bacteriuria: 2019 Update by the Infectious Diseases Society of America. Clin Infect Dis.

[10] ZalmanoviciTrestioreanu A, Lador A, Sauerbrun-Cutler MT, Leibovici L (2015). Antibiotics for asymptomatic bacteriuria. Cochrane Database Syst Rev.

[11] Kistler CE, Beeber AS, Zimmerman S, Ward K, Farel CE, Chrzan K, Wretman CJ, Boynton MH, Pignone M, Sloane PD. Nursing Home Clinicians’ Decision to Prescribe Antibiotics for a Suspected Urinary Tract Infection: Findings From a Discrete Choice Experiment. J Am Med Dir Assoc. 2020;21(5):675–682.e1. 10.1016/j.jamda.2019.12.004 PMC8121197 31974065

[12] Dyar OJ, Pagani L, Pulcini C (2015). Strategies and challenges of antimicrobial stewardship in long-term care facilities. Clin Microbiol Infect.

[13] Sommer-Larsen SD, Arnold SH, Holm A, Olesen JA, Cordoba G (2021). Quality of the Diagnostic Process, Treatment Decision, and Predictors for Antibiotic Use in General Practice for Nursing Home Residents with Suspected Urinary Tract Infection. Antibiotics.

[14] Carter RR, Montpetite MM, Jump RLP (2017). Mixed-Methods Pilot Study to Assess Perceptions of Antimicrobial Stewardship in Nursing Homes. J Am Geriatr Soc.

[15] van Buul LW, van der Steen JT, Doncker SMMM, Achterberg WP, Schellevis FG, Veenhuizen RB, Hertogh CMPM (2014). Factors influencing antibiotic prescribing in long-term care facilities: a qualitative in-depth study. BMC Geriatr.

[16] Schweizer AK, Hughes CM, Macauley DC, O’Neill C (2005). Managing urinary tract infections in nursing homes: a qualitative assessment. Pharm World Sci.

[17] Arnold SH, Jensen JN, Bjerrum L, Siersma VD, Bang CW, Kousgaard, MB, Holm A. Effectiveness of a tailored intervention to reduce antibiotics for urinary tract infections in nursing home residents: a cluster, randomised controlled trial. Lancet Infect Dis. 2021;21(11):1549–56. 10.1016/S1473-3099(21)00001-3 34303417

[18] Loeb M, Brazil K, Lohfeld L, McGeer A, Simor A, Stevenson K, Zoutman D, Smith S, Liu X, Walter SD (2005). Effect of a multifaceted intervention on number of antimicrobial prescriptions for suspected urinary tract infections in residents of nursing homes: Cluster randomised controlled trial. BMJ.

[19] Marshall S, Harrison J, Flanagan B (2009). The teaching of a structured tool improves the clarity and content of interprofessional clinical communication. BMJ Qual Saf.

[20] May C, Finch T (2009). Implementation, embedding, and integration: an outline of Normalization Process Theory. Sociology.

[21] May CR, Mair F, Finch T, MacFarlane A, Dowrick C, Treweek S, Rapley T, Ballini L, Ong BN, Rogers A, Murray E, Elwyn G, Légaré F, Gunn J, Montori VM (2009). Development of a theory of implementation and integration: Normalization Process Theory. Implement Sci.

[22] Pope C, Ziebland S, Mays N (2000). Qualitative research in health care: analysing qualitative data. BMJ.

[23] Fleming A, Bradley C, Cullinan S, Byrne S (2015). Antibiotic prescribing in long-term care facilities: a meta-synthesis of qualitative research. Drugs Aging.

[24] Lim CJ, Kwong MW, Stuart RL, Buising KL, Friedman ND, Bennett NJ, Cheng AC, Peleg AY, Marshall C, Kong DC (2014). Antibiotic prescribing practice in residential aged care facilities–health care providers’ perspectives. Med J Aust.

[25] Chan AJ, O’Donnell D, Kaasa B, Mathers A, Papaioannou A, Brazil K, Paraschiv N, Goldstein M, Sadowski CA, Dolovich L (2021). Barriers and facilitators of implementing an antimicrobial stewardship intervention for urinary tract infection in a long-term care facility. Can Pharm J (Ott).

[26] Potter R, Campbell A, Ellard DR, Shaw C, Gardner E, Agus A, O’Reilly D, Underwood M, Loeb M, Stafford B, Tunney M, Hughes C (2019). Multifaceted intervention to Reduce Antimicrobial Prescribing in Care Homes: a process evaluation of a UK-based non-randomised feasibility study. BMJ Open.

[27] Røsstad T, Garåsen H, Steinsbekk A, Håland E, Kristoffersen L, Grimsmo A (2015). Implementing a care pathway for elderly patients, a comparative qualitative process evaluation in primary care. BMC Health Serv Res.

[28] Björkman I, Berg J, Viberg N, Lundborg CS (2013). Awareness of antibiotic resistance and antibiotic prescribing in UTI treatment: A qualitative study among primary care physicians in Sweden. Scand J Prim Health Care.

[29] Overbeck G, Davidsen AS, Kousgaard MB (2016). Enablers and barriers to implementing collaborative care for anxiety and depression: a systematic qualitative review. Implement Sci.

[30] McEvoy R, Ballini L, Maltoni S, O’Donnell CA, Mair FS, Macfarlane A (2014). A qualitative systematic review of studies using the normalization process theory to research implementation processes. Implement Sci.

[31] Sutton E, Herbert G, Burden S, Lewis S, Thomas S, Ness A, Atkinson C (2018). Correction: Using the Normalization Process Theory to qualitatively explore sense-making in implementation of the Enhanced Recovery After Surgery programme: "It’s not rocket science". PLoS One.

[32] Burau V, Carstensen K, Fredens M, Kousgaard MB (2018). Exploring drivers and challenges in implementation of health promotion in community mental health services: a qualitative multi-site case study using Normalization Process Theory. BMC Health Serv Res.

