# Supplementary material for: Implementing an intervention to reduce use of antibiotics for suspected urinary tract infection in nursing homes – a qualitative study of barriers and enablers based on Normalization Process Theory
Source: BMC Geriatr. 2022 Mar 31;22:265. doi: 10.1186/s12877-022-02977-w (PMC8969390; doi:10.1186/s12877-022-02977-w)
Supplement: Supplementary file 1 — Additional file 1. [file 12877_2022_2977_MOESM1_ESM.docx]

**Additional file 1. Questions from the interview guide**

*The interview began with a few questions about the educational session: Overall experience, comprehensibility of content, perception of key messages, attitudes to key messages plus reasons for agreeing or not agreeing with key messages. Then came questions about the reflection tool (see below). Finally, came some questions about the communication tool. These were fewer in number and focused on frequency of use as well as reasons for use or non-use, comprehensibility, difficulties in using the tool, and consequences of using the tool in terms of perceived changes in communication with the GP.*

**The reflection tool**

**Coherence**

How were you introduced to the tool? (If the informant had not participated in the educational session)

What was your initial reaction to the tool?

Did the tool make sense to you? In what way? Why? Why not?

Did the tool (along with information from the educational session) seem to suggest that you should do something different in situations where a resident might have UTI?

What information did you receive about the purpose of the tool? (Probe on perceived sufficiency of information)

What information did you receive about how to use the tool? (Probe on perceived sufficiency of information)

Were there any parts of the tool which were difficult to understand? + Point to the tool and ask about specific sections and words that might be considered difficult (What did you do to increase your understanding of the tool?)

**Cognitive participation**

Who was responsible for implementation?

What did the person(s) responsible for implementation do to promote implementation?

What role did management play in the implementation of the tool?

(For staff responsible for implementation: What did management do to support your work with promoting the implementation of the tool?)

How did your colleagues react to using this tool? (Probe on experiences)

How often did you use the tool in daily practice? Did you use the tool every time you suspected UTI? (+ Probe: Why? / Why not?)

How were new employees introduced to the tool?

Have you noticed any differences between the various wards concerning the level of use? (Probe for further descriptions and explanations)

**Collective action**

Which staff used the tool?

What was it like to use the tool in interactions with residents? (Probe on challenges and how they were handled) Did using the tool require extra time to use? (In what way? Why?)

How did your collaborative partners outside the nursing home react to you using this new approach? (e.g., dementia nurses, psychiatry, and general practice) (How did you handle it if approaches differed?)

How did the residents react to the new approach to UTI? (Did they notice? Were there any negative reactions? If, so how did you handle it?)

How did the relatives react to the new approach to UTI? (Did they notice? Were there any negative reactions? If, so how did you handle it?)

**Reflexive monitoring**

Have you changed anything in your approach to UTI due to using the tool? (Or due to the information presented at the educational session?) (Probe about any individual or collective changes)

Did using the tool change your perception about when to conclude that it is likely that a resident has UTI? (How? / In what way?)

Did use of the tool change your perception about when to contact the GP in cases of suspected UTI? (How? / In what way?)

Did your use of the tool change over time? (How? / Why?) (Do you still use the tool? Why not?)
